# Editorial: Targeting DNA repair and the DNA damage response: Beyond the standard PI3 kinase-like kinases

**DOI:** 10.3389/fonc.2022.1023500

**Published:** 2022-09-27

**Authors:** John J. Turchi, Katherine S. Pawelczak, Michael Weinfeld, Peter J. McHugh

**Affiliations:** ^1^ Department of Medicine, Indiana University School of Medicine, Indianapolis, IN, United States; ^2^ NERx Biosciences, Indianapolis, IN, United States; ^3^ Department of Oncology, Cross Cancer Institute, University of Alberta, Edmonton, AB, Canada; ^4^ Department of Oncology, MRC Weatherall Institute of Molecular Medicine, University of Oxford, Oxford, United Kingdom

**Keywords:** DNA damage response (DDR), cancer therapy, small molecule inhibitor, DNA repair, synthetic lethality, drug discovery

Targeting DNA repair pathways and the DNA damage response (DDR) for cancer therapy has gained increased attention since the advent of PARP inhibitors and the demonstration of their clinical utility in BRCA-deficient cancers (1). In the subsequent 15 years, a major focus has been on development of kinase inhibitors targeting the PI3 kinase-related kinases (PIKKs) DNA-PK, ATM and ATR, to target genome stability and DNA replication stress inherent to many cancers, and there are excellent reviews of these efforts (2). In this collection we move beyond the standard PIKKS and present a series of primary research articles and focused reviews on non-PIKK targets and pathways within the DDR and DNA repair space. These represent the future of novel agents and targets and hold considerable potential to not only delineate mechanisms of basic molecular processes in DDR and repair but also as potential targets and therapeutics for the treatment of cancer. **Figure 1** highlights the breadth of targets and below we provide a brief summary of the review articles and primary research papers that make up this collection.

**Figure 1:**
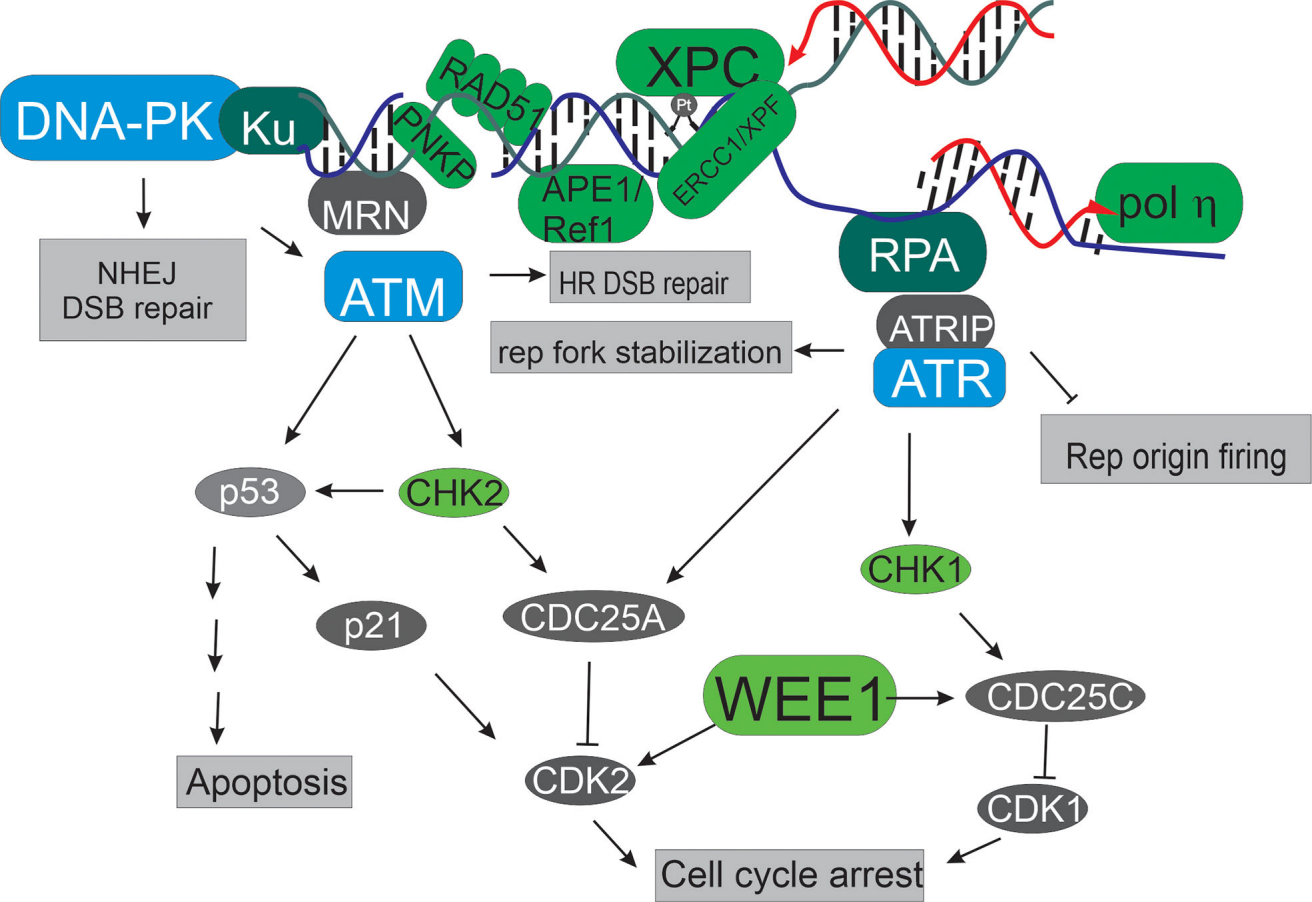
Targets in the DNA damage response and DNA repair pathways. The PIKKs, depicted in blue occupy central roles in the DDR. Beyond these targets, additional kinases are viable and interesting including Wee1, and the checkpoint kinases CHK1 and 2 (depicted in light green). Upstream of all these events are a series of proteins that often interact with DNA to fulfill their roles in the DDR and DNA repair (depicted in dark green). These include MRN, ERCC1/XPF, RPA and Ku that sense different DNA structures. Other potential targets include those that detect altered chemistry like APE1, and XPC (depicted in light green). A number of viable targets are involved in specific DNA metabolic events including Metnase, pol ƞ, and Rad51.

The review by Kelm et al. notes a variety of reasons for targeting proteins beyond the PIKKs: (i) their potential involvement in the repair of mitochondrial DNA as well as nuclear DNA; (ii) their role in protecting telomeres; (iii) the potential to expand the range of clinically beneficial synthetic lethal relationships; and (iv) the stimulation of the innate immune response when DNA repair is inhibited. The small molecule inhibitors detailed within this review target proteins in each of the four double strand break (DSB) repair pathways including Ku70/80, Artemis, DNA Ligase IV, PNKP, MRN complex, RPA, RAD51, RAD52, ERCC1-XPF, helicases, and DNA polymerase θ. For most of the compounds described, inhibition is based on either direct inhibition of enzymatic activity or disruption of protein-DNA or protein-protein interactions. While most of the inhibitors have IC_50_ values in the micromolar range, and therefore require further development, a few, such as the arylpyrazolone carboxylic acid-based Ku inhibitors (based on DNA-PK kinase activity) and the DNA polymerase θ inhibitor ART558, have IC50 values in the low nanomolar range. ART4215 (an undisclosed) derivative of ART558 is now undergoing Phase 1/2 clinical trials as a monotherapy or in combination with the PARP inhibitor talazoparib in patients with advanced or metastatic solid tumors, exhibiting the high potential benefit for such agents in a clinical setting.

## Targeting DNA binding proteins

Small molecule inhibitors of the RAD51 protein were initially described over a decade ago (3) and while useful tool compounds for research in some cases, translation to the clinic has been limited to compounds that target the Rad51 pathway through an unknown mechanism. In this collection, Gu et al. describe a novel class of compounds targeting Rad51 and present evidence for a direct Rad51 interaction and modulation of the cellular homologous recombination (HR) pathway. Intriguing evidence of single agent anticancer activity and in combinations in cell culture models will ultimately need to be verified *in vivo* to enable translation to the clinic.

Upstream of Rad51 in HR and ATR in the DDR lies Replication protein A (RPA), a trimeric factor that binds single-stranded DNA with high affinity, protecting these regions from nucleolytic degradation during DNA replication, repair and recombination, but also simultaneously controlling these processes through specific interactions with the actors involved. Interfering with the role of RPA in ssDNA protection has, therefore, the potential to perturb the DDR and leave ssDNA vulnerable to lethal degradation; consistently prior genetic studies imply that RPA ‘exhaustion’ can be lethal in cancer cells. VanderVere-Carozza et al. have previously developed a series of molecules that block RPA association with ssDNA (RPAi). In the current issue, they explore the selectivity of these compounds, demonstrate cellular toxicity across a range of cancer cells lines and show that stressed replication forks undergo degradation in the presence of RPAi. Moreover, RPAi synergy with therapeutically relevant DNA damaging agents are reported, as are antitumor effects in mouse xenograft models.

Metnase, whose name reflects a dual role as a methyltransferase and nuclease, is a factor produced through gene fusion and only found in primates. As reviewed by Nickoloff et al. Metnase plays a role in multiple DNA transactions, including in DSB repair processes, promoting NHEJ, where both the methyltransferase and nuclease domains contribute to its activity. Roles for Metnase in combating tumor replicative stress and a role mediating resistance to Topoisomerase II poisons also argue for the development of Metnase inhibitors. Nickoloff et al. also discuss the potential of EEPD1 structure-selective endonuclease inhibition in cancer. This factor plays a role in processing damaged replication forks and in promoting HR, and a case can be made for EEPD1 inhibition to target tumor vulnerabilities in the DDR and inherent replication stress, as well as a strategy for enhancing tumor chemosensitivity.

## Targeting kinases outside the PIKK family

Continuing with the theme of non-PIKK related targets, Bukhari et al. review the state-of-the-art in Wee1 inhibition. Wee1 is a tyrosine kinase originally identified by virtue of its key role in regulating the timing of *S. pombe* cell entry into mitosis, by phosphorylating and restraining the activity of CDK1. The role of Wee1 extends to the G2/M DNA damage checkpoint, and because many cancer cells harbor defects in the G1 checkpoint they become highly dependent upon the G2/M checkpoint – when the G2/M checkpoint is perturbed in such cells they will often enter a (terminal) mitotic catastrophe. Targeting Wee1 is also a potentially attractive therapeutic strategy due to emerging evidence regarding synthetic lethal interactions with DNA damage response regulators and because of potential synergies with radio- and chemotherapeutics. Bukhari et al. also describe the dozens of Wee1 inhibitor clinical trials performed to date, which suggest some promising results.

The checkpoint kinases have also been the subject of intense study with a number of inhibitors discovered over the years. Vaughan et al. in a primary research article identified the DDR and specifically Chk1 and 2 proteins as vulnerabilities in TCS-2 mutant renal cancers. This work highlights the complexity of pathway crosstalk and utility of chemical biology screening to elucidate previously unknown interactions. Impressive *in vivo* data are presented that demonstrate that abrogation of Chk1/2 activity with the dual AZD inhibitors results in prolonged tumor stasis and a reduction of cysts common in mTOR-driven disease. The question of whether specific Chk1 or Chk2 inhibitors recapitulate the results with the dual inhibitor remains but offer intriguing possibilities for these complex diseases.

Polynucleotide kinase/phosphatase (PNKP) is another very interesting kinase target but is responsible for phosphorylating DNA 5’-termini as opposed to proteins. This kinase activity along with intrinsic DNA 3’-phosphatase activity of PNKP is critical in the repair of DNA strand breaks to prepare DNA termini for ligation. This is especially important in the context of IR- induced DNA damage, where chemical modification of both bases and sugars often give rise to termini that are unable to be ligated. In this collection Sadat et al. describe a novel polysubstituted imidopiperidine PNKP 3’-phosphatase inhibitor encapsulated in a novel nano-particle formulation. Extensive analyses presented indicate excellent pharmacokinetics, biodistribution and *in vivo* efficacy. The *in vivo* experiments conducted in a colorectal tumor xenograft model demonstrate convincing radiosensitization and effective tumor reduction in the nano-particle formulation compared to the free-soluble drug, an effect that was demonstrated to be a function of enhanced bio-distribution and tumor uptake. Together these data position PNKP as a viable druggable target that is poised for further translation to clinical utility in treating cancer.

## Novel targets within the NER and BER pathways

Moving beyond DSB repair and kinases to nucleotide excision repair (NER) and base excision repair (BER) and crosslink repair, the manuscript by Weilbeer et al. presents additional *in silico* screening of a previously reported ERCC1-XPF inhibitor that focused on modifications of a specific side chain, resulting in a substantial increase in potency. The structure selective endonuclease ERCC1-XPF plays a role in repairing damage induced by crosslinking agents like platinum-based chemotherapies and ionizing radiation, and inhibition of its biological activity has the potential to potently sensitize cells to DNA damaging based therapies. The compounds discovered disrupt the protein-protein interaction required for heterodimerization, presenting an innovative mechanism of action for inhibition of ERCC1-XPF endonuclease activity. A lead hit was further evaluated and shown to sensitize cells to UV irradiation, cyclophosphamide crosslinking and ionizing radiation, further suggesting the potential for therapeutic applications with this family of novel inhibitors.

Nasrallah et al. examine the role that XPC may play in hematologic and non-dermatologic solid tumors. They point out that in addition to its canonical participation in the NER pathway, data strongly indicate XPC’s involvement in the BER, double strand break repair and interstrand crosslink repair pathways, possibly serving as a global DNA damage sensor. The authors go on to discuss the evidence associating XPC mutations, single-nucleotide polymorphisms and epigenetic alterations with elevated risk of malignancies as well as clinical response to chemotherapy. Based on these observations the authors recommend further investigation of XPC’s potential as a prognostic and/or predictive biomarker.

Continuing the BER theme, Mijit et al. investigated the influence of RelA (nuclear factor NF-κB p65 subunit) on the response of Kras-mutated pancreatic ductal adenocarcinoma (PDAC) cells to inhibitors of the redox function of Ref-1 (also known as the DNA repair endonuclease, APE1). While the BER activity of APE1 is targetable, the redox function has been shown to be important for cancer survival. They observed that RelA deficiency rendered the PDAC cells more resistant to the Ref-1 inhibitors. Furthermore, Ref-1 inhibition led to a marked reduction in IL-8, FOSB, and c-Jun, but this required the presence of active RelA. Their data indicate a critical role for RelA in redox homeostasis of Kras-mutated PDAC cells with implications for therapy targeting PDAC drug resistance.

## Targeting DNA damage tolerance pathways

While repair of DNA damage has garnered considerable attention, tolerance of damage remains an important component of how cells respond and cope with genetic abnormalities. Ler and Carty present a comprehensive review of DNA damage tolerance and the implication for carcinogenesis and opportunities for impinging on this pathway to treat cancer. The two main tolerance pathways discussed include translesion synthesis catalyzed by the by-pass polymerases and homology directed tolerance. The discussion of pathway choice offers unique insights into how cells coordinate the response to damage in relation to tolerance. Existing small molecule inhibitors of translesion polymerases are also reviewed and highlight the opportunities to disrupt this pathway to subvert cancer growth and resistance.

Drug development work targeting DNA repair associated polymerases is a rapidly growing field, and POLH represents a novel target within this family that has clinical promise due to its intriguing biological role in translesion synthesis that result in cellular resistance to damage from agents such as UV light and cisplatin. The work described by Wilson et al. presents a fragment-based drug development (FBDD) approach that utilizes a crystallization screen, resulting in novel x-ray crystal structures of small drug-like compounds bound to POLH. This emerging methodology and subsequent structural data have the potential to drive the rapid discovery and development of novel drug-like molecules, and may be particularly useful in developing drugs targeting complex enzymes like those in the polymerase families.

Clearly, the DDR space remains incredibly active in both discovery, preclinical and clinical development, with over 35 ongoing clinical trials spanning a variety of agents and targets. Importantly, there is an expanding wealth of knowledge regarding novel targets, therapeutic combinations and genetic alterations that are ripe for exploitation to impact the treatment of cancer with DDR targeted therapies. The long and circuitous route to clinical success of PARP inhibitors has provided a solid framework by which to pursue and evaluate the current and future DDR targeted agents, not least in the appropriate design of clinical trials. These experiences should remind us to not lose site of the underlying biology nor be swept up by the latest wave of success. The clinical reality is that the majority of those diagnosed with cancer will succumb to the disease. Only by pursuing the discovery and development of novel therapeutic strategies and targets can we expand the armamentarium to better equip our medical oncologist colleagues to impact the lives of cancer patients.

## Author contributions

JT, KP, MW and PM participated in the preparation of the initial manuscript draft and the final product. All authors contributed to the article and approved the submitted version.

## Funding

This work was supported by Cancer Research UK Program Award A24759 and MRC grant MR/X000192/1 to PM and NIH awards R01CA257430 and R01CA247370 to JT.

## Conflict of interest

Author KP was employed by NERx Biosciences.

The remaining authors declare that the research was conducted in the absence of any commercial or financial relationships that could be construed as a potential conflict of interest.

## Publisher’s note

All claims expressed in this article are solely those of the authors and do not necessarily represent those of their affiliated organizations, or those of the publisher, the editors and the reviewers. Any product that may be evaluated in this article, or claim that may be made by its manufacturer, is not guaranteed or endorsed by the publisher.
